# The effects of completion of continuum of care in maternal health services on adverse birth outcomes in Northwestern Ethiopia: a prospective follow-up study

**DOI:** 10.1186/s12978-022-01508-5

**Published:** 2022-10-08

**Authors:** Muluwas Amentie Zelka, Alemayehu Worku Yalew, Gurmesa Tura Debelew

**Affiliations:** 1 Department of Public Health, College of Health Sciences, Assosa University, Assosa, Ethiopia; 2 Department of Reproductive Health, School of Public Health, College of Health Sciences, Addis Ababa University, Addis Ababa, Ethiopia; 3 Department of Biostatistics and Epidemiology, School of Public Health, College of Health Sciences, Addis Ababa University, Addis Ababa, Ethiopia; 4 Department of Population and Family Health, Institute of Health, Jimma University, Jimma, Ethiopia

**Keywords:** Assosa town, Benishangul Gumuz, Continuum of care, Maternal health, Neonatal death, Perinatal death, Stillbirth, Ethiopia

## Abstract

**Background:**

Globally, around 4 million babies die within the first month of birth annually with more than 3 million stillbirths. Of them, 99% of newborn deaths and 98% of stillbirths occur in developing countries. Despite giving priority to maternal health services, adverse birth outcomes are still major public health problems in the study area. Hence, a continuum of care (CoC) is a core key strategy to overcome those challenges. The study conducted on the effectiveness of continuum of care in maternal health services was scarce in developing countries and not done in the study area. We aimed to assess the effectiveness of continuum of care and determinants of adverse birth outcomes.

**Methods:**

Community and health facility-linked prospective follow-up study designs were employed from March 2020 to January 2021 in Northwestern Ethiopia. A multistage clustered sampling technique was used to recruit 2198 pregnant women. Data were collected by using a semi-structured and pretested questionnaire. Collected data were coded, entered, cleaned, and analyzed by STATA 14. Multilevel logistic regression model was used to identify community and individual-level factors. Finally, propensity score matching was applied to determine the effectiveness of continuum of care.

**Results:**

The magnitude of adverse birth outcomes was 12.4% (95% CI 12.2–12.7): stillbirth (2.8%; 95% CI 2.7–3.0), neonatal mortality (3.1%; 95% CI 2.9–3.2), and neonatal morbidity (6.8%; 95% CI 6.6–7.0). Risk factors were poor household wealth (AOR = 3.3; 95% CI 1.07–10.23), pregnant-related maternal complications during pregnancy (AOR = 3.29; 95% CI 1.68–6.46), childbirth (AOR = 6.08; 95% CI 2.36–15.48), after childbirth (AOR = 5.24; 95% CI 2.23–12.33), an offensive odor of amniotic fluid (AOR = 3.04; 95% CI 1.37–6.75) and history of stillbirth (AOR = 4.2; 95% CI 1.78–9.93). Whereas, receiving iron-folic acid (AOR = 0.44; 95% CI 0.14–0.98), initiating breastfeeding within 1 h (AOR = 0.22; 95% CI 0.10–0.50) and immunizing newborn (AOR = 0.33; 95% CI 0.12–0.93) were protective factors. As treatment effect, completion of continuum of care via time dimension (β = − 0.03; 95% CI − 0.05, − 0.01) and space dimension (β = − 0.03; 95% CI − 0.04, − 0.01) were significantly reduce perinatal death.

**Conclusions:**

Adverse birth outcomes were high as compared with national targets. Completion of continuum of care is an effective intervention for reducing perinatal death. Efforts should be made to strengthen the continuum of care in maternal health services, iron supplementation, immunizing and early initiation of breastfeeding.

## Plain Language Summary

Adverse birth outcomes are a major public health problem and a big challenge in Ethiopia, particularly in the study area. They encompass stillbirth, neonatal death, and neonatal illness within 28 days after birth. Globally, about 4 million babies die within the first month of birth annually with more than 3 million stillbirths. Of these, about 99% of newborn deaths and 98% of stillbirths occur in developing countries. As a solution to overcome those problems, a continuum of care in maternal health services is a core strategy. Therefore, this study was planned to determine how effective continuum of care in maternal health service is in reducing perinatal death and factors contributing to the adverse birth outcomes. In this study, 2198 pregnant women were recruited and followed for 11 months. The health condition of women was frequently assessed and recorded during pregnancy, childbirth and the period until 42 days after childbirth, as well as the health condition of the babies until 28 days after the birth, the package of maternal health services received, and adverse birth outcomes. Among the 2198 pregnant women enrolled in the study, 248 women encountered adverse birth outcomes (52 had stillbirths, 58 had neonatal death and 138 had neonatal illness). Risk factors of adverse birth outcomes were a poor household wealth index quintile, pregnancy-related maternal complications, offensive odor amniotic fluid, and history of stillbirth. On the other hand, protective interventions against adverse birth outcomes were receiving iron supplementation during pregnancy, initiating breastfeeding within 1 h, and immunizing the newborn. Moreover, completions of continuum of care in maternal health services via time and space dimensions reduced perinatal death. In conclusion, neonatal and perinatal deaths were high in the study areas. Completions of continuum of care in maternal health services via time and space dimensions reduced perinatal death, neonatal death, and stillbirth. The results of this study can inform national health policymakers, maternal and child programmers, and other stakeholders to prioritize and strengthen protective intervention and continuum of care in maternal health services.

## Background

Women play an important role in the child rearing and family management, but their deaths from maternity-related causes is a significant social, economic and personal disasters [1]. Globally, around 4 million babies die within the first month of birth with more than 3 million stillbirths. Hence, 99% of newborn deaths occur in developing countries [2]. It is a big challenge for the world population and is still a public health problem, particularly in developing and low-income countries [3].

In Africa, annually 1 million stillbirths occur, of these, 300,000 cases occur during labour and childbirth. One million babies die within the first month, of those, half a million die within the first day. In Sub-Saharan Africa, the under-five child mortality rate is 16 times higher as compared with the rate in the high-income countries due to poor access to quality services and discontinuation of care in maternal health services [2, 4]. The deaths peak during the time of birth, the first day, and the first week of life.

The health needs of the mother and newborn are inseparably linked together [5, 6]. In the past decade: maternal, newborn, and child health policies and programs addressed their health needs separately; this gap and challenge in care provision, especially newborn health, are subjected to disadvantages. To overcome those challenges, a continuum of care (CoC) in maternal health services is one of the key strategies [6]. Thus, it reaches mothers and babies at a crucial time and place to reduce the risk of death, illness, or disability in the neonate [7, 8]. The health of newborns relies on the good linkage of maternal health intervention because newborn health is a sensitive indicator of the continuity of maternal health services [6–8]. Despite this, almost half of African women are not receiving skilled care during childbirth and postnatal care services after delivery [4].

Existing evidence show that when the utilization rate of essential maternal health services reach 90%, neonatal death can be reduced by 37–67% (430,000–800,000 of neonatal deaths) [4]. Hence, the continuum of care in maternal health services via time dimension can reduce neonatal mortality: completion of continuity of Antenatal (ANC) services can reduce 7–14%; continuity of care at childbirth can reduce 19–34% and continuity of postnatal (PNC) services can reduce 10–27% of neonatal deaths. Similarly, space dimension continuity of care can avert neonatal death: continuity of care at family/community level interventions (14–32%); outpatient/outreach interventions (7–14%), and clinical setting interventions (26–51%) of neonatal death can avert [6, 7]. A variety of evidence reveal that completion of continuity of care in maternal health services via time and space dimensions can reduce adverse birth outcomes (stillbirth, neonatal mortality, and morbidity) [4, 9–15].

Despite priority given to maternal health services, adverse birth outcomes (stillbirth, neonatal death, and illness) are still major public health problems in Ethiopia, particularly in the Benishangul Gumuz Region. However, majority of the existing studies in the region are cross-sectional in design and facility-based survey which can’t show a cause and effect relationship. Besides, they use the ordinary binary logistic and linear regression that may have a limitation in identifying factors at different levels and may not properly examine the treatment effect of interventions. Thus, in this study, clear epidemiological data on the adverse birth outcomes (stillbirth, neonatal death, and morbidity) were generated using a prospective follow-up study design, which was found to be important evidence for the implementation of maternal health services in the country Ethiopia in general and in Benishangul Gumuz Region in specific.

Therefore, the objective of this study was to investigate the effect of completion of continuum of care in maternal health services on adverse birth outcomes using propensity score matching (PSM) and multilevel logistic regression models.

## Methods

### Study design and setting

Community and health facility-linked prospective follow-up study was conducted in Benishangul-Gumuz Regional State (BGRS) from March 2020 to January 2021. The region is one of the eleven regional states of Ethiopia. Assosa town is the capital city of the region, located at 670KMs to the Western of Addis Ababa, the capital city of Ethiopia. The region has three zones, three city administration, twenty-one districts/*Woredas*, one special district/*Woreda’s*, and 475 *Kebele’s* (439 rural and 36 urban). The region represents around 4.6% of the total land area of Ethiopia and most of the people in the region are sparsely populated [16]. The total population of the region in 2022 was 1,219,017 and female in reproductive age group was 328,324 [17].

### Source population and study participants

All pregnant women and births that registered as live births, as well as stillbirths at the time of birth within the follow-up period, were considered as source populations. Whereas, the study population were newborns that registered as “live births” or “stillbirths” (which is declared by women, birth attendants, or health workers) at the time of birth and selected by simple random sampling techniques.

### Sample size and sampling technique

The sample size for this study was calculated using STATA/MP 13.0 software by considering two population proportion formulas. The outcome variable was the adverse birth outcomes (stillbirth, neonatal death, and any illness within the neonatal period) and completion of the continuum of care in maternal health services was considered as exposure (predictor) variable for the adverse birth outcomes. There is no similar study in Ethiopia that examined the effect of the continuum of care in maternal health services on adverse birth outcomes; a study conducted in Uttar Pradesh, India was used to estimate the minimum required sample size [13]. Accordingly, the proportion of adverse birth outcomes, “*neonatal death”*, among mothers who use a complete continuum of care in maternal health services is estimated to be 4.29% (*P*_1_ = 0.0429), and the proportion of adverse birth outcomes, “*neonatal death”*, among mothers who never use maternal health services is estimated to be 8.43% (*P*_2_ = 0.0843) [13]. A 95% confidence level and 80% power were used to detect a 4.14% difference. In addition, a ratio (r) of 1:1 was considered for the exposed and unexposed groups. Then, the pooled population proportion (P) = $\frac{{\mathrm{P}}_{1}+{\mathrm{P}}_{2}}{1+\mathrm{r}}$ was calculated (**P** = 0.0636). Finally, a design effect of 2 and a non-response rate of 10% were considered. Based on these assumptions, the final sample size was found to be 2402 pregnant women.

Since this research work was carried out at a regional level, the study subjects (pregnant women) were chosen using a multistage clustered sampling technique. The sampling procedure used for this study was as follows: primarily two zones and one town/city administration were chosen by simple random sampling (SRS). Following that, four districts/“*woredas*” from the Assosa Zone, two districts/“*woredas*” from the Metekel Zone, and two districts/“*woredas*” from the Assosa town/city administration were chosen by simple random sampling (SRS) technique. Thirdly, from each selected district/“*woreda*”, seven kebeles (except Assosa district/“*woreda*”: 10 kebeles and Assosa town administration: five ketenas) were selected and included in the study. Then, among the selected kebeles/*ketenas* (7 kebeles from each district/“*woreda*”, 10 kebeles from Assosa district/“*woreda*” and five ketenas from each district/“*woreda*” of town/city administration), pregnant women were enumerated by using house-to-house visit and all obtained and registered pregnant women were included in the study.

BSC Midwifery and Health Extension Workers (HEWs) assessed and diagnosed pregnancy status of the women. All women who claimed 8 weeks or longer pregnancy, as determined by the loss of two consecutive menses and pregnancy screening criteria (S_1_), were considered for eligibility and joined the study, which was followed for 11 months. Assuming that each household with pregnant women had at least one pregnant woman, households with pregnant women and neonates were selected as the final sampling unit (*FSU*). Meantime, all health facilities found within the catchment areas were listed and considered as a candidate for the health facility-based survey. Therefore, 46 health facilities (3 hospitals, 12 health centers, and 31 health posts) were found within the catchment areas and included in the health facility-based survey.

The inclusion criteria were births that were registered or informed as live births or stillbirth after the expulsion of placenta and whose mother was a permanent resident of the sampled areas. Whereas, pregnant women with hearing or other communication disabilities, severely ill and mentally ill women, pregnant women whose pregnancy is less than 8 weeks, and pregnant women who had completed fourth ANC visit at the time of the baseline survey were excluded.

### Data collection process

Data collection was conducted using semi-structured questionnaires and registration format adapted from EDHS 2011 [18], National Technical Guidance for Maternal and Perinatal Death Surveillance and Response (MPDSR) 2017 [19], MCH Program Indicator Survey 2013 [20], and survey tools conducted in Jimma Zone, Southwestern Ethiopia [21], Rural Southern Ethiopia [22] and other relevant different kinds of literature. The instrument was prepared in English and translated into the local language (“*Amharic*”) and then back-translated to English to ensure the validity of the instrument. Following that, training was offered for data collectors and supervisors for 3 days, and also pre-test was carried out on 35 individuals, located outside of the study areas/cluster.

During actual data collection, the principal investigator and supervisors were frequently supervising and checking the work of data collectors, and also clarification and direction were forwarded to those who had doubts. Moreover, chronbach alpha at 0.7 cut-off point was used to test inter item consistency of the indicators to measure the composite score of adverse birth outcomes, continuum of care and household wealth index quintile.

### Measurement of outcomes and interventions

Adverse birth outcome: pregnant women who experienced a pregnancy termination after 28 weeks of gestational age, categorized as “*stillbirth*,” or neonates who showed any evidence of life after complete expulsion or extraction from their mother and had any illness within 28 days, categorized as “*neonatal morbidity,*” or neonates who died before 28 days after delivery, categorized as “*neonatal mortality*.”

Continuum of care in maternal health services: package of interventions consisting of a composite measure of nine variables (1st ANC, 2nd ANC, 3rd ANC, and 4th ANC, Skill delivery care, 1st PNC, 2nd PNC, 3rd PNC and 4th PNC services). Pregnant women who miss at least one or more packages of intervention/s categorized as *discontinuation of care*, otherwise, receive the entire recommended minimum package of interventions considered as “*completing the continuum of care in maternal health services.*”

Intervention or exposure group: pregnant women who used the entire maternal health services (ANC, SD, and PNC) in a continuous manner were considered as “*exposure groups*” or “*completion of the continuum of care in maternal health services*. ”

Control or non-exposure group: pregnant women who missed at least one service in maternal health services (ANC, SD, and PNC) were considered as “*non-exposure groups*” or “*discontinuation of care in maternal health services.*”

### Data management and analysis

Data were coded and entered into Epi. Info version 7.2.2.6 to develop skipping patterns and avoid logical mistakes. Then, data were cleaned, edited, and analyzed using STATA Software version 14. All variables were computed for descriptive statistics. Analysis with only one independent variable was performed; the crude odds ratio and 95% confidence interval were used to select candidate variables for multivariable analysis at p < 0.25. At the level of significance (p < 0.05), a maximum likelihood estimate of the independent effect on the outcome variable was calculated. The household wealth index was calculated and categorized by using Principal Component Analysis (PCA).

Before running the full model, effect modification or interaction effect at p < 0.1 and multi-collinearity effect between independent variables using variance inflation factors (VIF > 10%) were assessed. All independent variables included had VIF < 10 and the multi-collinearity effects of each variable were p < 0.1. Hence, there was no significant interaction and the multi-collinearity effects were detected.

Since the sampling procedure for this study was a multistage clustered sampling procedure; due to cluster variability multilevel logistic regression model was applied to detect determinant factors of adverse birth outcomes (stillbirth, neonatal death, and any neonatal illness). Thus, for this study, ‘Kebeles/Ketenas’ were considered as clusters, and cluster level variables such as place of residence, access to the hospitals, access to the health centers, access to the health posts, and household wealth index were taken as level-2 variables. Women who gave birth during follow study were nested within their household wealth index and the community. As a result, women’s individual-level variables were socio-demographic, obstetric, information, maternal health services, and newborn health services were taken as level-1 variables. Log likelihood ratio (LR) test was performed to confirm the goodness of fit for the multilevel model that was found to be statistically significant indicating that the dataset is a best fit to the model.

Finally, the effect of continuum of care in maternal health services on perinatal death was estimated by Propensity Score Matching (PSM). The treatment effects were measured by Average Treatment Effect in Treated (ATT) with β and 95% CI at p < 0.05.

## Results

### Response rate

A total of 2439 pregnant women were enrolled and considered for the study. Of them, 241 pregnant women were lost to follow-up from the study for a variety of reasons, making a response rate of 91.5%. After excluding lost-to-follow-up and incomplete data, a total of 2198 total deliveries were recorded and completed during a follow-up study period. Finally, 2065 mothers’ birth outcomes were included in the final analysis by excluding the abortion cases.

### Newborn characteristics and health-related issues

The characteristics of newborns born were: 1102 (53.7%) were male and 359 (17.4%) were amniotic members broken before labour started. More than three-fourths of women, 1585 (76.8%), reported that the color of amniotic fluid was clear, whereas 305 (14.8%) of women stated that amniotic fluid had a bad odor. The majority of women, 1896 (91.8%), mentioned that their babies were ever breastfed within 28 days, and 1851 (84.2%) of women stated that the newborns cried immediately after birth (S_2_).

### Adverse birth outcomes (stillbirths, neonatal morbidity, and neonatal mortality)

Even though a total of 2065 pregnant women gave birth, 248 (12.4%; 95% CI 12.2–12.7) were accompanied by adverse birth outcomes (stillbirth 52 (2.8%; 95% CI 2.7–3.0), neonatal morbidity 138 (6.8%; 95% CI 6.6–7.0) and neonatal death, 58 (3.1%; 95% CI 2.9–3.2).

After weighing for cluster variation, among stillbirths, 24 (39.7%) took place in the hospital, followed by the health center 20 (37.4%), and also 38 (64.3%) were attended by midwifery/nurse/health officers. The major possible causes of stillbirth were maternal infection/sepsis 38 (80.7%), maternal malnutrition 15 (41.2%), and unnecessary medication 12 (17.8%). The top three pregnancy-related problems during pregnancy before the occurrence of stillbirth were severe abdominal pain 31 (66.2%), excessive vaginal bleeding 23 (50.1%), and blurred vision 11 (31.6%). Whereas, the common illnesses that women encountered during pregnancy before the occurrence of stillbirths were febrile illness 25 (62.0%), malnutrition 7 (13.3%), and anemia 5 (13.0%).

Among newborns who died within the postnatal period, 35 (59.5%) died within 48 h and 43 (72.9%) of neonatal deaths were classified as early neonatal deaths. The three major neonatal characteristics that caused neonatal death: birth asphyxia 31 (48.9%), sepsis/infection 18 (33.6%) and prematurity of birth 7 (13.9%). Similarly, the three major maternal characteristics that caused neonatal death were obstructed labour 30 (56.4%), obstetric sepsis/maternal infection 29 (54.7%), and antepartum hemorrhage (APH) 16 (33.3%) (Table 1).

**Table 1:** Adverse birth outcome and related factors among the study subject in Benishangul Gumuz Region, Northwestern Ethiopia, March 2020–January 2021

Variables	Frequency	Un-weighted %	Weighted %
Adverse birth outcome			
No	1817	87.0	87.6
Yes	248	12.0	12.4
Births encountered by stillbirth			
No	2013	97.5	97.2
Yes	52	2.5	2.8
Place of stillbirth takes place (*n* = 52)			
Hospital	24	46.2	39.7
Health center	20	38.5	37.4
Health post/clinic	5	9.6	13.0
Home	3	5.8	9.9
Attendant of stillbirth (*n* = 52)			
Midwifery/Nurse/HO	38	73.1	64.3
Medical Doctor	6	11.5	12.8
HEW	5	9.6	13.0
Family member	3	5.8	9.9
Possible cause of stillbirth (*n* = 52) (multiple response)			
Infections/sepsis	38	73.1	80.7
Maternal malnutrition	15	28.8	41.2
Unnecessary medication^a^	12	23.1	17.8
Any illness during pregnancy before the event (*n* = 52) (multiple response)			
Fever/febrile illness	25	48.1	62.0
Malnutrition	7	13.5	16.3
Anemia	5	9.6	13.0
Epilepsy/convulsion	5	9.6	13.0
High blood pressure	2	3.8	3.4
Pregnancy-related problems before the event (*n* = 52) (multiple response)			
Severe abdominal pain	31	59.6	66.2
Excessive vaginal bleeding	23	44.2	50.1
Severe headache	13	25.0	18.4
Blurred vision	11	21.2	31.6
Foul-smelling vaginal discharge	9	17.3	25.2
Shortness of breathing	7	13.5	16.7
Swelling of fingers, face, and leg	5	9.6	13.0
Neonatal health outcomes within 28 days			
Neonatal well being	1817	90.3	90.1
Neonatal morbidity	138	6.9	6.8
Neonatal mortality	58	2.9	3.1
Age at which neonatal death occurred (n = 58)			
Within 2 days	35	60.3	59.5
2–7 days	8	13.8	13.4
8–28 days	15	25.9	27.1
Classification of neonatal death			
Early neonatal death	43	74.1	72.9
Late neonatal death	15	25.9	27.1
Neonatal characteristics cause neonatal death			
Asphyxia	31	53.5	48.9
Neonatal infections/sepsis	18	31.0	33.6
Prematurity	7	12.1	13.9
Others	2	3.4	3.6
Maternal characteristics suspected risk factors for neonatal death (multiple response)			
Obstructed labour	30	51.5	56.4
Obstetric sepsis	29	50.0	54.7
APH	16	27.6	33.3
Ruptured uterus	9	15.5	15.3
Preeclampsia/eclampsia	8	13.8	17.4

^a^Women receive either overdoses of drugs or un-prescribed medications or self-medications

### Prevalence of perinatal and neonatal mortality rate

Of the study subjects, a total of 2013 live births, 52 stillbirths, 43 early neonatal deaths, and 15 late neonatal deaths. As a result of cluster variation, weighted estimation was used for each parameter. Thus, weighted analysis was done to avoid over-estimation and under-representation and it was found to be the rate of stillbirth was 28 (95% CI 27, 30) per 1000 births, neonatal mortality rate was 31 (95% CI 29, 32) per 1000 live births (early neonatal death was 23 (95% CI 21, 24) and late neonatal death was 8 (95% CI 8, 9) per 1000 live births). The weighted perinatal mortality rate was 50 (95% CI 49, 52) per 1000 births (Table 2).

**Table 2:** Status of perinatal and neonatal mortality in Benishangul Gumuz Region, Northwestern Ethiopia, March 2020–January 2021

Indicators	Frequency	Rate/1000 (95% CI) (un-weighted)	Rate/1000 (95% CI) (weighted)
Total births	2065		
Total live births	2013		
Stillbirth	52	25 (19–33)	28 (27–30)
Early neonatal mortality	43	21 (16–24)	23 (21–24)
Late neonatal mortality	15	7 (4–12)	8 (8–9)
Perinatal mortality	95	46 (37–56)	50 (49–52)
Neonatal mortality	58	29 (22–37)	31(29–32)

### Factors affecting adverse birth outcome

The determinant factors of adverse birth outcomes were assessed using the multilevel logistic regression model. Before running the full model, ICC (ρ) was calculated in the empty model for the outcome to decide whether the data fit the multilevel model or not. Then, ICC (ρ) was calculated as a full model for the outcome to detect the variability attributed to clusters after controlling the individual level.

Likewise, the adverse birth outcomes, ICC (ρ) were calculated in the empty model and it was found to be 0.32, indicating that 32% of the variation was contributed by cluster variations. The test of the preference of log-likelihood *versus* logistic regression was statistically significant (P < 0.0001). Then, the full model was run by including both the cluster-level and individual-level variables and the ICC (ρ) was increased to 0.53. This again indicated that 53% of the variation was attributed to cluster-level variables. The preferences of log-likelihood versus logistic regressions were determined which indicates that statistically significant (p < 0.0001). Hence, this is suggested that using multilevel analysis is crucial rather than a logistic regression model (Table 3).

**Table 3:** Parameters of odds ratio and test of goodness-of-fit of the mixed-effects multilevel models, Benishangul Gumuz Region, Northwest Ethiopia, 2021

Models	Fixed intercept-cons (95% CI)	Random effect as level-2 variance var(-cons (95% CI))	Intra-class correlation coefficient: ICC(ρ)	Log-likelihood (LR)-deviance	Significance of LR test vs. logistic regression (P-value)
Adverse birth outcomes^a^					
Empty model	0.08 (0.05, 0.12)	1.55 (0.89, 2.71)	0.32 = 32%	− 670.72	P < 0.0001
Full model	0.1 (0.002, 2.92)	3.75 (1.74, 8.06)	0.53 = 53%	− 220.11	P < 0.0001

*P* value less than 0.05 is statistically significant and the data fit for the multilevel model

^a^Multilevel regression model applied to measure the effect of factors on outcome

After adjusting for confounding effects, a multivariable multilevel logistic regression model was performed. Then, the determinant factors of adverse birth outcomes are identified at the cluster and individual-level factors such that at the cluster-level (*level-2*) factors: household wealth index and at individual-level (*level-1*) factors: women’s educational status, history of abortion, time of 1st ANC initiation, iron-folic acid supplementation, initiate breastfeeding within one hour, pregnant-related problems during labour and immediately after childbirth, presence of an offensive odor of amniotic fluid and immunizing newborn within the postnatal period were found to have a statistically significant association with adverse birth outcomes.

The odds of having adverse birth outcomes among women who resided in a poor household wealth index (AOR = 3.3; 95% CI 1.07, 10.23) were three times higher than among women who resided in a rich household wealth index. Similarly, the odds of having adverse birth outcomes among women who attended high school (AOR = 6.12; 95% CI 1.87, 20.1) were six times higher than among women who had no formal education.

Women initiated a 1st ANC visit between 4 and 6 months of gestational age (AOR = 0.38; 95% CI 0.18, 0.8), received iron-folic acid supplementation during pregnancy (AOR = 0.44; 95% CI 0.14, 0.98), initiate breast feeding within 1 h (AOR = 0.22; 95% CI 0.10, 0.50) and immunized newborn within the postnatal period (AOR = 0.33; 95% CI 0.12, 0.93) were lower to have adverse birth outcomes than among women with their counterpart.

Women who had pregnant-related complications during pregnancy (AOR = 3.29; 95% CI 1.68, 4.46), labour and childbirth (AOR = 6.08; 95% CI 2.36, 15.48) and immediately after childbirth (AOR = 5.24; 95% CI 2.23, 12.33) were higher to have adverse birth outcomes as compared with their counterpart. Similarly, women who had an offensive odor of amniotic fluid (AOR = 3.04; 95% CI 1.37, 6.75) were three times higher to have adverse birth outcomes than women who didn’t have an offensive odder of amniotic fluid (Table 4).

**Table 4:** Multilevel models analysis on factors associated with adverse birth outcome (stillbirth, neonatal death, and neonatal illness) Benishangul Gumuz Region, Northwest Ethiopia 2021

Determinant factors	Adverse birth outcomes		Crud OR 95% CI	Adjusted OR 95% CI
	No	Yes		
Level-2 (community level) variables				
Place of residents				
Urban	613 (85.5)	104 (14.5)	1	1
Rural	1204 (89.32)	144 (10.68)	1.12 (0.55, 2.27)	2.17 (0.48, 9.85)
Time takes to reach HC				
< 2 h	1362 (88.16)	183 (11.84)	1	1
≥ 2 h	455 (87.50)	65 (12.50)	**1.98 (1.15, 3.42)**	0.96 (0.32, 2.85)
Household wealth index				
1st quintile (poor)	566 (84.60)	103 (15.40)	1.63 (0.97, 2.74)	**3.3 (1.07, 10.23)**
2nd quintile (middle)	604 (85.67)	101 (14.33)	**1.91 (1.22, 3.0)**	2.20 (0.98, 5.0)
3rd quintile (rich)	647 (93.63)	44 (6.37)	1	1
Leve-1 (individual level) variables				
Age (years)				
< 20	138 (86.79)	21 (13.21)	1	1
20–29	1173 (88.86)	147 (11.14)	0.79 (0.46, 1.36)	0.50 (0.04, 6.27)
≥ 30	506 (86.35)	80 (13.65)	0.89 (0.5, 1.6)	0.35 (0.03, 4.67)
Ethnicity				
Berta	989 (90.32)	106 (9.68)	1	1
Others	828 (85.36)	142 (14.64)	0.98 (0.58, 1.66)	0.96 (0.3, 3.12)
Woman education level				
No formal education	1146 (89.25)	138 (10.75)	1	1
Primary school	333 (86.05)	54 (13.95)	1.41 (0.95, 2.1)	1.41 (0.5, 3.97)
High school	180 (83.33)	36 (16.67)	1.52 (0.96, 2.43)	**6.12 (1.87, 20.1)**
Tertiary education	158 (88.76)	20 (11.24)	0.79 (0.45, 1.37)	0.91 (0.22, 3.77)
Partner education level				
No formal education	1033 (89.21)	125 (10.79)	1	1
Primary school	199 (82.23)	43 (17.77)	**2.08 (1.32, 3.29)**	1.49 (0.53, 4.16)
High school	245 (88.45)	32 (11.55)	0.99 (0.62, 1.61)	1.44 (0.52, 4.07)
Tertiary education	286 (86.93)	43 (13.07)	0.84 (0.54, 1.29)	0.69 (0.22, 2.22)
History of stillbirth				
No	1263 (90.15)	138 (9.85)	1	1
Yes	130 (76.02)	41 (23.98)	**3.46 (2.18, 5.5)**	**4.2 (1.78, 9.93)**
Information on MHS				
No	150 (81.97)	33 (18.03)	1	1
Yes	1667 (88.58)	215 (11.42)	**0.62 (0.38, 0.99)**	0.93 (0.3, 2.89)
Place of delivery for previous delivery				
Home	354 (86.76)	54 (13.24)	1	1
Health post	322 (91.48)	30 (8.52)	**0.82 (0.44, 1.51)**	1.19 (0.46, 3.09)
Health center	531 (86.91)	80 (13.09)	**0.59 (0.37, 0.97)**	1.26 (0.55, 2.89)
Hospital	186 (92.54)	15 (7.46)	**0.33 (0.16, 0.71)**	0.39 (0.1, 1.55)
Availability of MHS				
No	103 (83.74)	20 (16.26)	1	1
Yes	1714 (88.26)	228 (11.74)	0.75 (0.42, 1.34)	2.76 (0.18, 42.85)
Provision of maternal health care for the community				
No	115 (80.99)	27 (19.01)	1	1
Yes	1702 (88.51)	221 (11.49)	0.59 (0.36, 1.0)	1.1 (0.1, 11.37)
Time of 1st ANC initiation				
1–3 months of GA	459 (84.84)	82 (15.16)	1	1
4–6 months of GA	1223 (92.37)	101 (7.63)	**0.31 (0.21, 0.46)**	**0.38 (0.18, 0.8)**
After 6 months of GA	81 (61.83)	50 (38.17)	**4.16 (2.41, 7.19)**	0.5 (0.11, 2.32)
Number of ANC visits				
< 4	523 (85.18)	91 (14.82)	1	1
≥ 4	1294 (89.18)	157 (10.82)	**0.69 (0.49, 0.97)**	0.98 (0.42, 2.3)
IFA supplementation				
No	306 (78.06)	86 (21.94)	1	1
Yes	1511 (90.32)	162 (9.68)	**0.31 (0.22, 0.44)**	**0.44 (0.14, 0.98)**
TT during pregnancy				
No	410 (81.03)	96 (18.97)	1	1
Yes	1407 (90.25)	152 (9.75)	**0.41 (0.29, 0.57)**	1.08 (0.41, 2.81)
Initiate BF within 1 h				
No	303 (71.97)	118 (28.03)	1	1
Yes	1514 (92.09)	130 (7.91)	**0.15 (0.11, 0.22)**	**0.22 (0.10, 0.50)**
Pregnant-related problems during pregnancy				
No	1525 (91.37)	144 (8.63)	1	1
Yes	292 (73.74)	104 (26.26)	**3.99 (2.86, 5.60)**	**3.29 (1.68, 6.46)**
Pregnant-related problems during childbirth				
No	1641 (92.71)	129 (7.29)	1	1
Yes	176 (59.66)	119 (40.34)	**10.42 (7.27, 14.9)**	**6.08 (2.36, 15.48)**
Husband decision making on health services				
No	777 (82.40)	166 (17.60)	1	1
Yes	1040 (92.69)	82 (7.31)	**0.42 (0.29, 0.58)**	0.73 (0.36, 1.48)
Duration of labour				
< 12 h	1346 (92.38)	111 (7.62)	1	1
B/n 12–24 h	369 (80.57)	89 (19.43)	**3.62 (2.52, 5.19)**	1.21 (0.56, 2.63)
> 24 h	102(68.0)	48 (32.0)	**6.29 (3.93, 9.96)**	0.73(0.22, 2.34)
Pregnant-related problems immediately after delivery				
No	1715 (91.86)	152 (8.14)	1	**1**
Yes	102 (51.52)	96 (48.48)	**12.15 (8.1, 18.31)**	**5.24 (2.23, 12.33)**
Time interval for 1st PNC				
Within 2 days	431 (85.52)	73 (14.48)	1	1
B/n 3–7 days	682 (90.45)	72 (9.55)	0.93 (0.59, 1.45)	0.68 (0.3, 1.53)
B/n 8–42 days	431 (89.23)	52 (10.77)	0.91 (0.5, 1.64)	0.75 (0.27, 2.04)
The time of PMRM before labour				
≤ 1 h	717 (88.08)	97 (11.92)	1	1
1–12 h	1002 (88.52)	130 (11.48)	**1.45 (1.03, 2.03)**	0.76 (0.39, 1.47)
> 12 h	73 (77.66)	21 (22.34)	**5.37 (2.8, 10.27)**	–
Bad odor of amniotic fluid				
No	1568 (89.14)	191 (10.86)	1	1
Yes	249 (81.37)	57 (18.68)	**2.26 (1.48, 3.46)**	**3.04 (1.37, 6.75)**
Immunized the newborn				
No	257 (73.43)	93 (26.57)	1	1
Yes	1560 (90.96)	155 (9.04)	**0.23 (0.16, 0.32)**	**0.33 (0.12, 0.93)**

The bold values indicate statistically significant association (*p* < 0.05)

### Effect of continuity of maternal health services on perinatal death

To reduce the potential of confounding effects, we utilized the propensity score matching (PSM) approach to compare women who had a complete continuum of care in maternal health services with their main interventions to those who had terminated/discontinued the services. Among the different distinct techniques of propensity score matching (PSM), one-to-one matching was utilized to quantify the effect of an intervention on perinatal death. After matching treated and controlled individuals, the effects of the completion of a continuum of care in maternal health services via time and space dimensions on perinatal death were determined.

Hence, the result found to be received 1st ANC visit (β = − 0.14; 95% CI − 0.21, − 0.07; p < 0.001); completed 4th ANC visit (β = − 0.05; 95% CI − 0.08, − 0.02; p < 0.001); skilled attendant of ANC services (β = − 0.04; 95% CI − 0.07, − 0.01; p = 0.006); completed continuity of care for both 4th ANC and skilled delivery (β = − 0.04; 95% CI − 0.07, − 0.02; p = 0.001); completion of continuum of care in maternal health services via time dimension (β = − 0.03; 95% CI − 0.05, − 0.01; p = 0.001); completed key services of ANC package (β = − 0.05; 95% CI − 0.07, − 0.03; p < 0.001), completion of key services of PNC package (β = − 0.07; 95% CI − 0.09, − 0.05; p < 0.001), complete whole key service maternal health services (β = − 0.05; 95% CI − 0.07, − 0.04; p < 0.001) and completed continuum of care via space dimension (β = − 0.03; 95% CI − 0.04, − 0.01; p = 0.003) were associated with a significant reduction in the likelihood of perinatal death.

After matching treated and control individual; average treatment effect of completion of continuum of care in maternal health services via time dimension were lower perinatal death by *0.03* as compared with discontinued maternal health services among the treated group (β = − 0.03; 95% CI − 0.05, − 0.01). Similarly, average treatment effect of completion of continuum of care in maternal health services via space dimension were lower perinatal death by *0.03* as compared with discontinued maternal health services among the treated group (β = − 0.03; 95% CI − 0.04, − 0.01). In other words, women who completed continuum of care in maternal health services had less perinatal death than those who that did discontinued the services by *0.03* among the treated group (Table 5).

**Table 5:** Propensity score matching analysis on the effect of a continuum of care in maternal health services on perinatal death Benishangul Gumuz Region, Northwest Ethiopia 2021

Factors	Perinatal death		ATE		ATET	
	No	Yes	*β* 95% CI^a^	*P*-value	*β* 95% CI^a^	*P*-value
I. Continuity of care in maternal health services via time dimension						
First ANC services						
No received	152 (83.1)	31 (16.9)				
Received	1818 (96.6)	64 (3.4)	− **0.14 (**− **0.21, **− **0.08)**	*P* < *0.001*	− **0.14 (**− **0.21, **− **0.07)**	*P* < *0.001*
Fourth ANC services						
Discontinued	561 (91.4)	53 (8.6)				
Completed care	1409 (97.1)	42 (2.9)	− **0.05 (**− **0.07, **− **0.02)**	*P* < *0.001*	− **0.05 (**− **0.08, **− **0.02)**	*P* < *0.001*
Delivery care services						
Unskilled delivery	736 (93.8)	49 (6.2)				
Skilled delivery	1234 (96.4)	46 (3.6)	− **0.04 (**− **0.06, **− **0.01)**	*P* = *0.002*	− **0.04 (**− **0.07, **− **0.01)**	*P* = *0.006*
Continuity of care for both 4th ANC and skilled delivery						
Discontinuity of services	945 (93.8)	64 (6.2)				
Completion of the services	1025 (96.9)	31 (3.1)	− **0.04 (**− **0.06, **− **0.02)**	*P* < *0.001*	− **0.04 (**− **0.07, **− **0.02)**	*P* = *0.001*
Completion of continuity of maternal health services (ANC, SD, and PNC)						
Discontinuity of COC	1258 (94.3)	76 (5.7)				
Completion of a COC	712 (97.4)	19 (2.6)	− **0.03 (**− **0.05, **− **0.01)**	*P* = *0.002*	− **0.03 (**− **0.05, **− **0.01)**	*P* = *0.001*
II. Continuity of care for key maternal health services						
Continuity of key services of ANC package						
Discontinuity of key services	855 (92.5)	69 (7.5)				
Completion of key services	1115 (97.7)	26 (2.3)	− **0.05 (**− **0.07, **− **0.02)**	*P* < *0.001*	− **0.05 (**− **0.07, **− **0.03)**	*P* < *0.001*
Continuity of key services of PNC package						
Discontinuity of key services	967 (91.8)	86 (8.2)				
Completion of key services	1003 (99.1)	9 (0.9)	− **0.07 (**− **0.09, **− **0.05)**	*P* < *0.001*	− **0.07 (**− **0.09, **− **0.05)**	*P* < *0.001*
Continuity of key services of all key services of the MHS package						
Discontinuity of key services	1211 (93.3)	87 (6.7)				
Completion of key services	759 (99.0)	8 (1.0)	− **0.05 (**− **0.07, **− **0.03)**	*P* < *0.001*	− **0.05 (**− **0.07, **− **0.04)**	*P* < *0.001*
III. Continuity of care for maternal health services via space dimension						
Discontinuity of care	1168 (93.7)	79 (6.3)				
Completion of a COC	802 (98.0)	16 (2.0)	− **0.03 (**− **0.05, **− **0.01)**	*P* = *0.001*	− **0.03 (**− **0.04, **− **0.01)**	*P* = *0.003*

The bold values indicate statistically significant association (*p* < 0.05)

^a^Adjusted for a place of residence, educational status, occupational status, household wealth index, and distance of health facility

## Discussion

### Status of adverse birth outcome

Adverse birth outcomes are the most essential vital data used to evaluate maternal and child health programs and plan evidence-based interventions. They serve as a gauge for the quality and effectiveness of maternal health services provided to mothers and children. The basic indicators used to measure adverse birth outcomes were stillbirth, neonatal death, and any illness during the postnatal period. Thus, this study found that 12.4% of women had adverse birth outcomes which were lower than evidence from Northern Ethiopia 27.5% [23]. This is because of the variation in study time and design. Due to the time variation, a community-based intervention implemented by HEW will reduce the magnitude of adverse birth outcomes in the study area.

According to the global target, every country will have 12 or fewer stillbirths per 1000 births by 2030. Even though developed countries and upper-middle-income countries were meet their target, developing and low-income countries will have to more than double present progress to reach this target [24]. Besides these, the magnitude of stillbirth was 28 stillbirths per 1000 births in the study area. It was much higher than the global target (12 per 1000 pregnancies), Mumbai Slums 8.68 per 1000 births [25], and Ghana 1.5% [26]. But, it was lower than the study done in Rural Pakistan 39.2 per 1000 birth [27], Northern Ethiopia 8.1% [23], Southwest Nigeria 4.7% [28], and Gondar University Hospital 7.1% [29]. However, this finding was consistent with evidence from rural southwestern Uganda 2.4% [30], Rural Uganda 2.4% [30], and the Tigray region (2.3%) [31]. The reason may be the reduction of stillbirth is much slower than in other countries due to poor utilization of recommended ANC services via visit-based and content-based continuity of care and also the quality of maternal health services was poor as compared with other countries.

Neonatal mortality is the probability of dying within the first month of life which is the most pertinent national indicator in maternal and child health programs. Hence, this study found that the neonatal mortality rate was 31 per 1000LBs. This finding is almost similar to the evidence from EDHS 2019 (33 per 1000LBs) [32], Ghana 30.85 per 1000LBs [33], and the Indian State of Bihar 32.2 per 1000LBs [34]. However, it was lower than evidence from Rural Pakistan 49.65 per 1000LBs [27], Tigray region 62.5 per 1000LBs) [35], and Jimma Zone 35.5 per 1000LBs [21]. But, it was much higher than the study in Mumbai Slums 13.98 per live births [25], Vietnam 11.65 per 1000LBs [36], India 12 deaths per 1000LBs [10], and the Tigray 4.6% [31]. This implies that neonatal mortality is still high in developing countries and Ethiopia, particularly in the study area and there is no improvement in the reduction of neonatal mortality to achieve the global and national targeted goal.

In this study, we found that the perinatal mortality rate was 50 per 1000 births. This finding is consistent with the pooled estimate of the perinatal mortality rate in Ethiopia was 51.3 per total birth [37]. However, this finding was higher than the study done in Hawassa town health facilities 2.7% [38] and rural northern Ghana 39 per 1000 births [39]. Whereas, it was lower than evidence from low-income countries 55 per 1000 births [40], Rural Pakistan 69.95 per birth [27], and Rural Congo 61 per 1000 births [41]. Among newborns that died within the postnatal periods, 59.5% of the newborns died within 48 h and early neonatal deaths contribute 72.9% of neonatal deaths. This finding is consistent with the different studies done in the country and outside the country [35, 36, 40, 42].

Worldwide, the major causes of neonatal death are infections (sepsis/pneumonia, tetanus, and diarrhea), prematurity/preterm birth, and birth asphyxia. In line with these, we found that the three major neonatal characteristics that caused neonatal death were birth asphyxia (48.9%), sepsis/infection (33.6%), and prematurity of birth (13.9%). This finding was supported by different studies [21, 25, 39–43]. However, some variation between countries may be sought because of the variability of quality, availability, and utilization rate of maternal and newborn health services.

Maternal complications during pregnancy and childbirth are predisposing factors for neonatal death. As result, safe motherhood is a proven initiative to prevent newborn death by overcoming pregnant-related complications [42]. Hence, this study found that obstructed labour (56.4%), obstetric sepsis/maternal infection (54.7%), and APH (33.3%) were common pregnant-related problems that cause neonatal death. This finding was consistent with evidence from low and middle-income countries which found that obstructed labor and placental abruption cause birth asphyxia, and also maternal infections cause neonatal infections that cause neonatal death [5]. This is due to maternal and neonatal health conditions being interlinked with each other.

In this study, the possible causes of stillbirth were maternal infection or obstetric sepsis, maternal malnutrition, and unnecessary medication. This finding is consistent with evidence from low-income countries [40] and rural northwest Bangladesh [44]. Moreover, before the occurrence of stillbirth, women frequently suffered from pregnant-related problems (severe abdominal pain, excessive vaginal bleeding, blurred vision, and any illness during pregnancy (febrile illness, malnutrition, and anemia). This finding is supported by another study [24]. This is because pregnant-related problems and maternal illness during pregnancy cause poor placental function, either with fetal growth restriction or preterm labor, or both, which is a common cause of stillbirths. As a result, different evidence argued that better prenatal care is critical to preventing 1.3 million stillbirths and ending unnecessary neonatal deaths [20].

### Determinant factors of adverse birth outcome

Among community-level factors, the household wealth index is a predictor of adverse birth outcomes. Besides, women who resided in a poor household wealth index were three times more likely to have adverse birth outcomes. This is because the level of the household index is directly related to access to information and receiving health services, which enable them to prevent themselves and their newborn from any illness and adverse event.

Educational status is a determinant factor of adverse birth outcomes, in which women who attended high school were six times more likely to have adverse birth outcomes as compared with women who had no formal education. This may be because educated people have better knowledge and information on the impact of adverse birth outcomes, as result; they early diagnose the problems and report properly for further treatment and management as compared with people with no formal education. Moreover, people who lack education did not report any adverse events and believed that the event was a usual life rather than considered health problems. So, the magnitude of adverse birth outcomes was high among educated people.

In this study, initiating 1st ANC visit between 4 and 6 months of gestational age reduced adverse birth outcomes by 62%. This study is consistent with the previous studies in Gondar University Hospital [29] and Ghana [26]. This could be the fact that during ANC visits, relevant health issues can be identified and addressed early. This is only feasible if pregnant women present themselves to a suitable health facility at an early stage of labor and pregnancy. As a result, the probability of occurring adverse birth outcomes will be remission.

According to World Health Organization (WHO) directives, IFA supplementation during pregnancy, early initiation of breastfeeding within 1 h for the newborn, and immunization increase the survival of newborn by overcoming adverse events. In line with these, we found that women who received IFA supplementation during pregnancy and initiate breastfeeding within 1 h for their newborn, and immunized newborns within the postnatal period were less likely to have adverse birth outcomes. This finding is concurrent with previous studies within the country and abroad [9, 13, 21, 35].

The occurrence of pregnant-related problems during pregnancy, childbirth, and immediately after childbirth decreases the survival of the newborns. Besides, we depicted that women who had pregnant-related complications during pregnancy, childbirth, and immediately after childbirth were more likely to have adverse birth outcomes. This finding is consistent with evidence from low and middle-income countries [45], Developing countries [46], Ghana [26], the Indian State of Bihar [34], Jimma Zone [21], and Gondar University Hospital [29]. This is the fact that women who have pregnant-related complications were prone to infection and birth asphyxia which are the leading cause of stillbirth, neonatal death, and neonatal illness. This is also explained by the health condition of newborns relying on maternal health status before, during, and after conception.

In principle, the amniotic fluid should be odorless or have a slightly sweet odor. However, if it is noticed that smells bad, it could be a sign and symptom of uterine infection. Thus, this study revealed that women who had an offensive odor of amniotic fluid were three times more likely to have adverse birth outcomes. This is explained by the offensive odor of amniotic fluid as an indication of infections, which is the leading cause of stillbirth birth, neonatal death, and illness during the postnatal period.

### Effects of a continuum of care in maternal health services on perinatal death

Maternal health interventions during pregnancy, childbirth, and after childbirth have a beneficial health impact on maternal and newborn health outcomes [47]. This is because early detection of pregnancy-related disorders throughout pregnancy, management of labor and delivery, care of any adverse event, and treatment of serious infectious infections and acute malnutrition are all effective interventions of maternal health services packages, which could reduce 77% of perinatal mortality [48].

In line with these, we found that women received 1st ANC visit; completed 4th ANC visit; skilled attendant of ANC services; completed continuity of care for both 4th ANC and skilled delivery; completion of a continuum of care in maternal health services; completed key services of ANC package, completion of key services of PNC package, complete whole key service maternal health service and completed continuum of care via space dimension were significantly reduced perinatal deaths. This finding is consistent with previous studies conducted in the country and abroad [9, 10, 21, 26, 27, 31, 41, 43, 49–61]. This is because more than one-third of all child deaths occur in the first month of life, providing skilled care to mothers during pregnancy, as well as during and after birth, greatly contributes to child survival, with two-thirds of newborn deaths preventable if known, effective health measures are provided at birth and during the first week of life.

## Conclusions

In this study, we concluded that neonatal and perinatal mortality rates were high as compared with national and international targets. Risk factors for adverse birth outcomes were poor household wealth index, pregnant-related maternal complications during pregnancy, childbirth, and immediately after childbirth, bad odor of amniotic fluid, and history of stillbirth. Whereas, the protective interventions were receiving iron and folic acid supplementation during pregnancy, initiating breast feeding within 1 h for newborn, immunizing newborn, and completing continuity of care in maternal health services via time and space dimensions. Efforts should be made to strengthen supplementation of iron and folic acid during pregnancy, immunizing newborns, and early initiation of breast feeding for the newborns as well as early detection and treatment of risk factors for poor birth outcomes were strongly recommended. Moreover, increased attention to the continuum of care in maternal health services, to women before and after giving birth, as well as newborn is required to effectively reduce adverse birth outcomes, perinatal, and neonatal mortality. This must be accomplished by reaching out to disadvantaged populations, particularly economically poor populations, and providing them with the necessary health care. Moreover, this study strongly recommended further study on the effectiveness of the continuum of care using the randomized clinical trial (RCT) and pregnancy test (HCG).

## Data Availability

The datasets used and analyzed during the current study are available from the corresponding author on reasonable request.

## Abbreviations

ANC: Antenatal care
AOR: Adjusted odd ratio
APH: Antepartum hemorrhage
ATE: Average treatment effect
ATET: Average treatment effect on the treated
BF: Breast feeding
BGRS: Benishangul Gumuz Region
CI: Confidence interval
CoC: Continuum of care
COR: Crude odd ratio
EDHS: Ethiopia Demographic and Health Survey
FSU: Final sampling unit
GA: Gestational age
HC: Health Center
HCG: Human chorionic gonadotropin
HEW: Health Extension Workers
HO: Health officer
ICC: Intra-class correlation coefficient
IFA: Iron folic acid
IRB: Institutional Review Board
LBs: Live births
LR: Log likelihood ratio
MCH: Maternal and child health
MHS: Maternal health services
MPDSR: Maternal and Perinatal Death Surveillance and Response
NMR: Neonatal mortality rate
PCA: Principal component analysis
PNC: Postnatal care
PSM: Propensity score matching
RCT: Randomized Clinical Trial
REC: Research Review and Ethics Committee
SPH: School of Public Health
SPSS: Statistical Package for Social Sciences
SRS: Simple random sampling
TT: Tetanus toxoid
WHO: World Health Organization
